# The mtDNA mutation spectrum in the PolG mutator mouse reveals germline and somatic selection

**DOI:** 10.1186/s12863-021-01005-x

**Published:** 2021-11-26

**Authors:** Kendra D. Maclaine, Kevin A. Stebbings, Daniel A. Llano, Justin C. Havird

**Affiliations:** 1 Department of Integrative Biology, The University of Texas at Austin, 2415 Speedway #C0930, Austin, TX 78712 USA; 2 Neuroscience Program, The University of Illinois at Urbana-Champaign, 405 North Mathews Avenue, Urbana, IL 61801 USA; 3 Beckman Institute for Advanced Science and Technology, 405 North Mathews Avenue, Urbana, IL 61801 USA; 4 Department of Molecular an Integrative Physiology, 524 Burrill Hall, MC-114, 407 South Goodwin Avenue, Urbana, IL 61801 USA

**Keywords:** mtDNA, mtDNA mutations, PolG, Mutation spectrum, Germline mutations, mtDNA selection, Protein hydrophobicity, mitochondrial theory of aging, ROS

## Abstract

**Background:**

Mitochondrial DNA (mtDNA) codes for products necessary for electron transport and mitochondrial gene translation. mtDNA mutations can lead to human disease and influence organismal fitness. The PolG mutator mouse lacks mtDNA proofreading function and rapidly accumulates mtDNA mutations, making it a model for examining the causes and consequences of mitochondrial mutations. Premature aging in PolG mice and their physiology have been examined in depth, but the location, frequency, and diversity of their mtDNA mutations remain understudied. Identifying the locations and spectra of mtDNA mutations in PolG mice can shed light on how selection shapes mtDNA, both within and across organisms.

**Results:**

Here, we characterized somatic and germline mtDNA mutations in brain and liver tissue of PolG mice to quantify mutation count (number of unique mutations) and frequency (mutation prevalence). Overall, mtDNA mutation count and frequency were the lowest in the D-loop, where an mtDNA origin of replication is located, but otherwise uniform across the mitochondrial genome. Somatic mtDNA mutations have a higher mutation count than germline mutations. However, germline mutations maintain a higher frequency and were also more likely to be silent. Cytosine to thymine mutations characteristic of replication errors were the plurality of basepair changes, and missense C to T mutations primarily resulted in increased protein hydrophobicity. Unlike wild type mice, PolG mice do not appear to show strand asymmetry in mtDNA mutations. Indel mutations had a lower count and frequency than point mutations and tended to be short, frameshift deletions.

**Conclusions:**

Our results provide strong evidence that purifying selection plays a major role in the mtDNA of PolG mice. Missense mutations were less likely to be passed down in the germline, and they were less likely to spread to high frequencies. The D-loop appears to have resistance to mutations, either through selection or as a by-product of replication processes. Missense mutations that decrease hydrophobicity also tend to be selected against, reflecting the membrane-bound nature of mtDNA-encoded proteins. The abundance of mutations from polymerase errors compared with reactive oxygen species (ROS) damage supports previous studies suggesting ROS plays a minimal role in exacerbating the PolG phenotype, but our findings on strand asymmetry provide discussion for the role of polymerase errors in wild type organisms. Our results provide further insight on how selection shapes mtDNA mutations and on the aging mechanisms in PolG mice.

**Supplementary Information:**

The online version contains supplementary material available at 10.1186/s12863-021-01005-x.

## Background

All animals contain mitochondrial DNA (mtDNA), which contains genes encoding oxidative phosphorylation (OXPHOS) proteins, tRNAs, and rRNAs necessary for mitochondrial gene translation. Mitochondrial genes, along with many nuclear genes, encode the proteins that make up the electron transport chain (ETC) which generates the majority of cellular energy in the form of ATP. Mutations in mtDNA can lead to significant metabolic dysfunction. Examples of pathological mtDNA mutations in human diseases include Kearns-Sayre syndrome, which causes myopathy, Myoclonic epilepsy with ragged red fibers (MERRF), a neurodegenerative disease, and Leber hereditary optic neuropathy (LHON), which results in blindness [1–4]. In an ecological context, variation in mtDNA sequences also has consequences for phenotypic variation and fitness in natural populations [5]. Variation in mtDNA can be particularly challenging to understand, as: 1) offspring inherit a heteroplasmic population of mtDNA genomes matrilineally, 2) mtDNA mutations increase with age through somatic mtDNA mutations, and 3) all mtDNA mutations arise as heteroplasmic variants [6–8].

Insight into the origins and consequences of mtDNA mutations has been improved by the PolG mouse model, which was originally created to test the mitochondrial theory of aging [9]. This theory states that an accumulation of mtDNA mutations over an organism’s lifetime is a significant contributor to aging [10, 11]. PolG mice have deficient exonuclease proofreading capabilities in polymerase gamma, the sole polymerase responsible for mtDNA replication, resulting in an abnormal accumulation of mtDNA mutations [9]. These progeroid mutator mice experience several pathological symptoms, including a ~ 50% reduction in lifespan, kyphosis (curvature of the spine), an enlarged heart and spleen, and alopecia [9, 12]. Interestingly, there is evidence that PolG mice do not display other normal aging hallmarks, including an increase in reactive oxygen species (ROS) or normal osteoarthritis. PolG mice also fail to show increased lifespan from a calorie restricted diet [13–15]. The impact of exercise on PolG mtDNA mutations and phenotype is controversial, with some studies finding a decrease in mtDNA mutations and reduction in the severity of PolG phenotypes with cardiovascular exercise [12, 16], but this finding has been unable to be replicated using non-pooled next-generation sequencing [7].

Though many studies have provided insight into the aging physiology of PolG mice, there has been comparatively little work done to characterize the specific types of mtDNA mutations in PolG mice. For reference, wild type mice have been observed with as little as one mtDNA mutation per mouse [17], while PolG mice have hundreds or thousands of mtDNA point mutations at varying frequencies [12, 16]. Mutation spectra (e.g., the types of mutations observed and their relative frequencies) have not been examined thoroughly in PolG mice. One study found high numbers of cytosine to thymine mutations, which is unsurprising given that such mutations are characteristic of replication errors and should be abundant in PolG mice [18]. C to T mutations are also overrepresented in wild type mice, and are asymmetrically overrepresented on the H-strand [19, 20]. In PolG mice, these C to T mutations also led to an increase in the hydrophobicity of proteins. However, this study was limited by a low sample size (*n* = 2) [18]. It has also been suggested that mtDNA mutations are lowest in the D-loop in PolG mice [18, 21], which is the region where replication originates, although other studies have noted that large indels are most common in this region [22, 23].

Characterizing mtDNA mutations in PolG mice not only provides further insight into mitochondrial aging mechanisms but also serves as a model for examining mtDNA selection. Typically, mtDNA selection is examined on a population level in order to have an adequate number of mtDNA variants [24, 25]. The rapid accumulation of mtDNA mutations in the PolG mouse creates many mtDNA variants that can be detected within a single individual, rather than having to use an entire population. In PolG mice, mtDNA selection occurs between organisms through the germline. PolG wildtype littermates that have a functioning polymerase do not display premature aging, though they may inherit up to 80% of the mtDNA mutation load of their mutant littermates [7, 9, 12]. The resilience of these wildtype littermates suggests that germline selection on mtDNA excludes the most harmful mutations from being passed on to the next generation. Selection on mitochondrial function and mtDNA variants during early gametogenesis and embryogenesis is critical and has even been invoked to explain the evolution of the sequestered germline [26]. mtDNA and the mitochondria themselves undergo multiple bottlenecks during early gamete and embryo development, and many have suggested that this amounts to an intense “selective sieve” that results in only the most fit mitochondrial genomes being passed to the next generation [27–30].

Beyond strong purifying selection on mtDNA in the germline, mtDNA selection can occur somatically within organisms on the tissue, cell, and organelle levels [31–35]. Heteroplasmy in mtDNA through somatic mutations can cause mitochondrial dysfunction within an individual. Dysfunction arising from somatic mtDNA mutations can be resolved by cells or tissue undergoing apoptosis [31–33]. Within a cell, individual dysfunctional mitochondria can be degraded by mitophagy [34, 35]. More fully characterizing somatic and germline mtDNA mutations in PolG mice may provide insights into selection on mtDNA variants within and among individuals as well as processes of aging related to mtDNA mutations.

Here, we use mtDNA-enriched next-generation sequencing data to explore the mutation spectrum in the PolG mouse. We predicted that C to T transitions stemming from replication errors would dominate the mutational landscape, but that the spectra of germline vs. somatic mutations and their effects on resulting protein products may differ due to selection within individuals and across generations.

## Results

### PolG mutation spectra pipeline

**Fig. 1:**
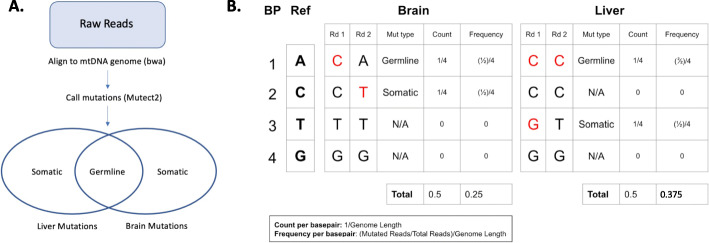
Methods to quantify abundance of mtDNA mutations. **A**. The pipeline used to call and classify mtDNA mutations **B**. The reference (Ref) shows a hypothetical 4 base pair mtDNA genome with 2 reads (Rd 1 and Rd. 2) aligned. Example mutation types, counts, and frequencies are displayed for two reads each from liver and brain tissue. Red bases highlight mutations. Mutations that occur in both tissues are germline, and mutations that occur in only one are somatic. Count defines numbers of unique variants, while frequency defines prevalence of a particular variant

Raw Illumina sequencing reads were aligned to the mouse mt genome. After mutations were called, they were separated into germline and somatic mutations (Fig. 1A). Mutations were also analyzed using two metrics: mutation count which evaluated whether a mutation was present at each reference basepair, and mutation frequency, which measures the prevalence of each mutation (Fig. 1B).

### PolG mtDNA mutations are more common in liver tissue

**Fig. 2:**
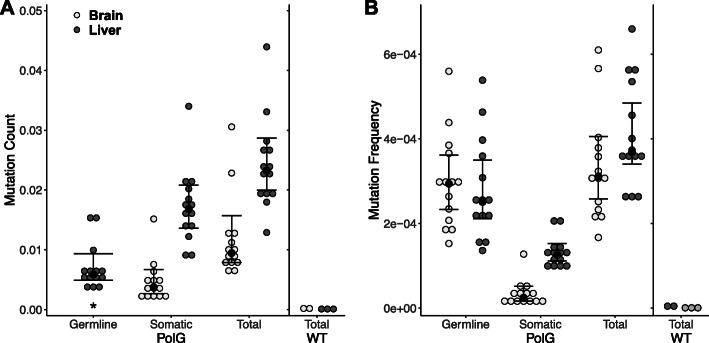
Germline (appearing in both tissues) and somatic (appearing only in one tissue) mutations in brain and liver tissue of PolG mice and liver tissue for wild type mice for both mutation count, **A**, and mutation frequency, **B**. Total is the sum of germline and somatic mutations. *N* = 14 for PolG, *N* = 3 for WT (wild type) liver, *N* = 2 for WT brain. PolG mice were between 9 and 12 months of age and WT mice were 9–10 weeks of age. Error bars are ±95% CI, black dots represent the median. *Because germline mutations are defined as being in both liver and brain tissue, tissue type cannot be separated for the mutation count metric, but it can be for the mutation frequency metric

**Fig. 3:**
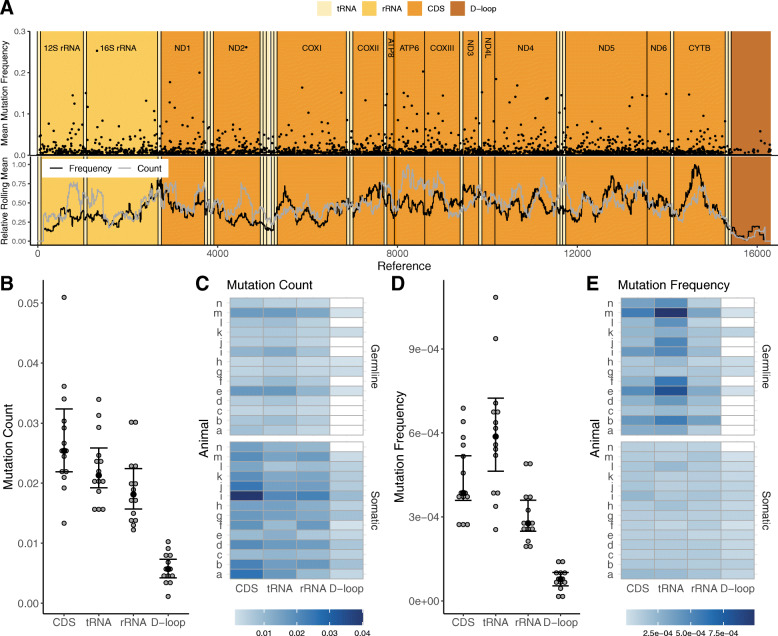
**A**. The mean mutation frequency is plotted across the entire mtDNA genome. Frequencies were averaged if more than one mutation appeared in one base pair. Normalized values were found using {(x/max(x))}. Lines show the rolling mean (250 bp) of both the frequency and count. Mutation Count, **B, C.** and Mutation Frequency, **D, E.** are shown for protein coding (CDS), tRNA, rRNA, and D-loop regions. Mutation count and frequency are normalized to region length. *N* = 14. Error bars are ±95% CI, black dots represent the median

**Fig. 4:**
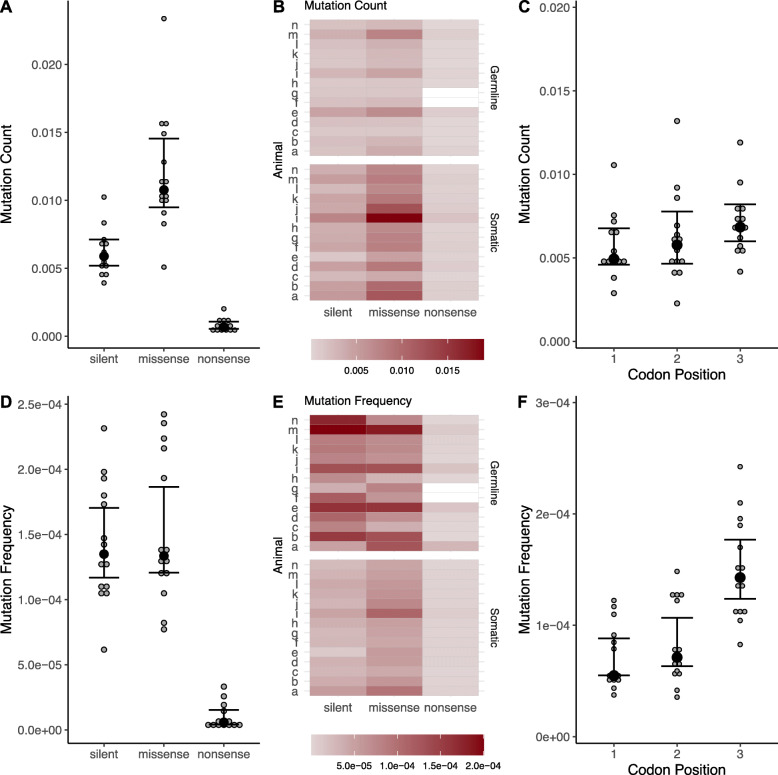
Mutation Count**, A, B**., and Mutation Frequency, **D, E**. are shown for silent, missense, and nonsense mutations in protein coding regions. The mutation count **C,** and frequency, **F,** are also shown for each codon position. *N* = 14. Error bars are ±95% CI, black dots represent the median

**Fig. 5:**
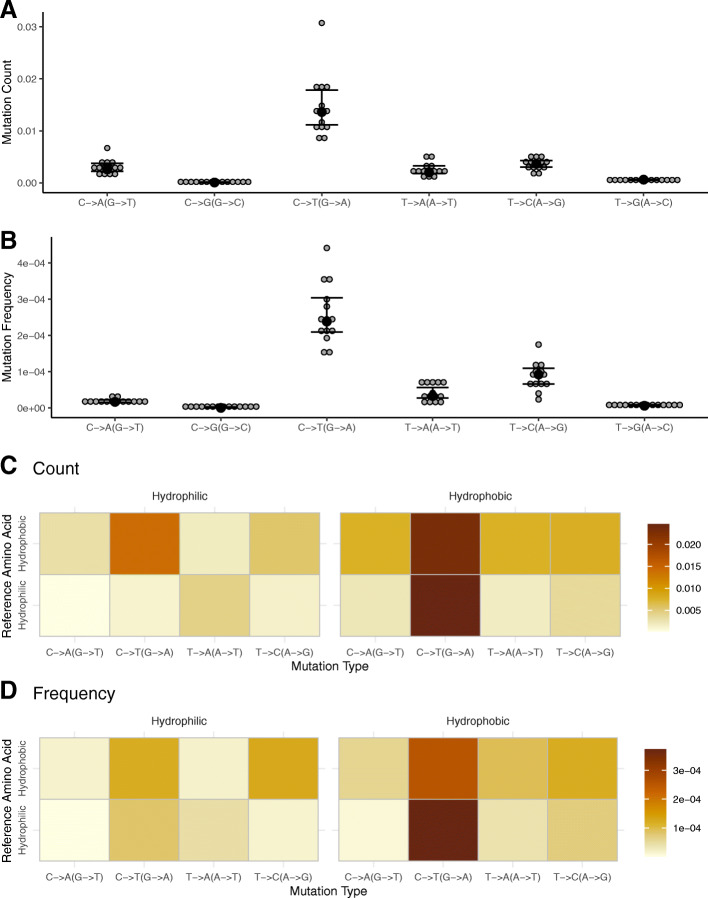
Mutation count, **A**., and Mutation frequency, **B.**, for each type of base pair substitution. *N* = 14. Error bars are ±95% CI, black dots represent the median. Heatmap showing types of base pair substitutions in missense mutations and their effects on amino acid hydrophobicity for **C.,** mutation count and **D.**, mutation frequency. The reference amino acid state is on the left side and the resulting variant is across the top. The four most common types of mutations are presented here (see Supplementary Figs. S6, S7 for other types) (Additional File 1)). Redundant changes (e.g., C- > T and G- > A) are combined. Only missense mutations are included in the heatmap

PolG mice accumulated many more mtDNA mutations compared to wild type mice (Fig. 2A, B). At an mtDNA coverage of about 10,000, only six point mutations were detected in the two brain samples, and four point mutations were found in the 3 liver samples from wild type mice (one fixed polymorphism compared to the reference genome was not included), while about 200 point mutations per PolG brain sample and 400 mutations per liver sample were found in the average PolG mouse. Importantly, the wild type mice used here were never introduced in the PolG line, unlike previous studies that included homozygous negative PolG mice as a control, which are sometimes referred to as “wild type” [12, 18, 36]. When all mutations were summed, mutation counts and frequencies were 106 and 24% higher respectively in liver tissue compared to brain tissue (Fig. 2A; *t* (13)=6.73, *p* < 0.001; Fig. 2B; *t* (13)=6.06, *p* < 0.001). Therefore, we only report on liver mutations in the main text, while analogous Figs. 3, 4, 5, and 7 from brain samples are presented in Supplementary Figures and showed the same trends (Supplementary Fig. 1–4, 5, 7, 8) (Additional File 1). In liver tissue, somatic mutation counts were about 1.5 times higher compared to germline mutations (Fig. 2A; *t* = 6.212, *p* = < 0.001) in PolG mice, but mutation frequencies were about 50% *lower* for somatic mutations (Fig. 2B; *t* = − 5.217, *p* < 0.001).

### Mutations are abundant throughout the mtDNA except in the D-loop

MtDNA mutations were detected over the entire mtDNA genome in PolG mice (Fig. 3A). MtDNA mutation frequency and mutation count tend to trend together, but there are regions where these measures appear to diverge (e.g., ATP6 and CYTB; Fig. 3A).

There were no significant differences in mutation counts among the tRNAs (Fig. 3B, C; *t =* − 1.855, *p* = 0.067) or rRNAs (Fig. 3B, C; *t =* − 0.460, *p* = 0.646) when compared to the protein coding regions (CDS), but the D-loop had 79% fewer mutations when compared with the CDS (Fig. 3B, C; *t* = − 11.805, *p* < 0.001). A similar pattern among the mtDNA genome regions was found in mutation frequency: The D-loop was lowest (Fig. 3D; *t* = − 14.226, *p* < 0.001) with an 82% lower average mtDNA mutation frequency when compared with the CDS, but unlike mutation count, the tRNA region had a 35% higher mutation frequency than the CDS (Fig. 3D, E; *t* = 4.421, *p* = < 0.001). There was a marginally significant negative interaction between mutation type (germline vs. somatic) and location because germline, but not somatic tRNA mutations tended to rise to high frequencies (Fig. 3E; *t* = − 2.007, interaction *p* = 0.048). Additionally, rRNA mutation frequency was 31% lower than the CDS region (Fig. 3E; *t* = − 2.679, *p* = 0.009). Most animals had no detectable D-loop germline mutations (Fig. 3C, E). Overall, these results suggest that the D-loop is depleted of mtDNA mutations in the PolG mouse, and the other regions have about the same number of mtDNA mutations, but frequency significantly varies among them.

### Missense mtDNA mutations are abundant, but rarely inherited

For the combined CDS, we evaluated how mutations affected the resulting amino acids. Nonsense mutations were rarest in both count and frequency (Fig. 4A; *t* = − 13.999, *p* < 0.001; Fig. 4D; *t* = − 14.922, *p* < 0.001). On average, missense mutations had twice the mutation count compared to silent mutations (Fig. 4A, *t* = 2.568, *p* = 0.013), but silent and missense mutations showed similar mutation frequencies (Fig. 4D; *t* = − 1.427, *p* = 0.158). For both mutation counts and mutation frequencies, there was a positive interaction between somatic/germline and silent/missense, such that somatic mutations were more likely to be missense than germline mutations (Fig. 4B, *t* = 2.889, interaction *p* = 0.005; Fig. 4E**;** *t* = 3.536, interaction *p* < 0.001).

There was no effect of codon position on mutation count (Fig. 4C; *t* = 1.479, *p* = 0.180, but there was a significant effect on mutation frequency (Fig. 4F; *t* = 17.160, *p* < 0.001), such that mutations in codon position 3 had 90% higher frequency compared with positions 1 and 2 (*p* < 0.001 for both). Overall, these results suggest that somatic mutations are more likely to be missense when compared to germline mutations, and though all three codon positions are equally likely to mutate, mutations in the third codon position of CDS regions rise to higher frequencies.

### C to T (G to A) transition mutations dominate the PolG mutation spectra, contributing to an increase in hydrophobic amino acids

There was a significant effect of base pair substitution type for both mutation count (Fig. 5A; *F* = 152, *p* < 0.001) and frequency (Fig. 5. B; *F* = 162, *p* = < 0.001). C to T (G to A) base pair transitions were the most abundant type of single base pair point mutation, showing 3 times higher mutation count and 2 times higher mutation frequency compared with T to C (A to G) mutations, the second most frequent base pair change (Fig. 5, 4A, B; *p* < 0.001 for all pairwise comparisons with C to T (G to A)). All other types of point mutations were also detected, although C to G (G to C) and T to G (A to C) mutations were exceedingly rare in our data (only 8 and 14 total mutations detected across all liver samples, respectively) and were not considered in analyses of amino acid changes.

Considering only missense mutations, those involving a change between hydrophilic and hydrophobic amino acids were the most common when examining amino acid properties (Supplementary Fig. S6, S7) (Additional File 1). In mixed linear models for mutation count and frequency that only include hydrophobic and hydrophilic changes in C to T mutations, there was no significant difference when the initial state of the amino acid was considered (Fig. 5C; *t =* − 2.018, *p =* 0.0523; Fig. 5D; *t =* − 0.242, *p =* 0.810) (i.e., hydrophilic and hydrophobic reference amino acids were equally likely to mutate), but in both mutation count and frequency, the mutated amino acid was more likely to be hydrophobic (Fig. 5C; *t =* 7.023, *p =* < 0.001; Fig. 5D; *t =* 6.145, *p =* < 0.001). Taken together, PolG mutations are primarily C to T (G to A) transitions which tend to increase the hydrophobicity of protein products.

### C to T strand asymmetry is absent after correcting for nucleotide bias

**Fig. 6:**
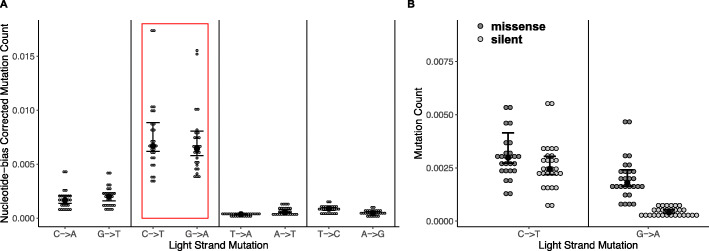
**A**. Strand asymmetry in mutation counts shown for each mutation, corrected for nucleotide bias (mutations that had very few changes were excluded). Only somatic mutations were included. **B.** Somatic mutation count is shown for missense and silent mutations for C to T and G to A changes. *N* = 14. Error bars are ±95% CI, black dots represent the median. Observing mutations on a strand level does not result in redundancy

We did not find a significant difference between somatic C to T and G to A point mutations after correcting for nucleotide bias on the L-strand (Fig. 6A; *F =* 0.446, *p =* 0.507), and the mean mutation count was approximately the same between the two mutation types. Using the L-strand as the reference and only including somatic mutations, there were about 30% more missense mutations on average than silent mutations for C to T changes (Fig. 6B; *F =* 2.155, *p* = 0.154). For G to A somatic mutations, there were three times more missense mutations than silent mutations ((Fig. 6B; *F =* 61.29, *p* < 0.001). Taken together, these data indicate that though there is no strand bias for PolG mutations after correcting for the nucleotide composition of the L-strand, there is a difference in the effect that L-strand and H-strand mutations have on amino acid sequences of mt-encoded proteins.

### Indels are less common than point mutations and tend to be small deletions in PolG mice

**Fig. 7:**
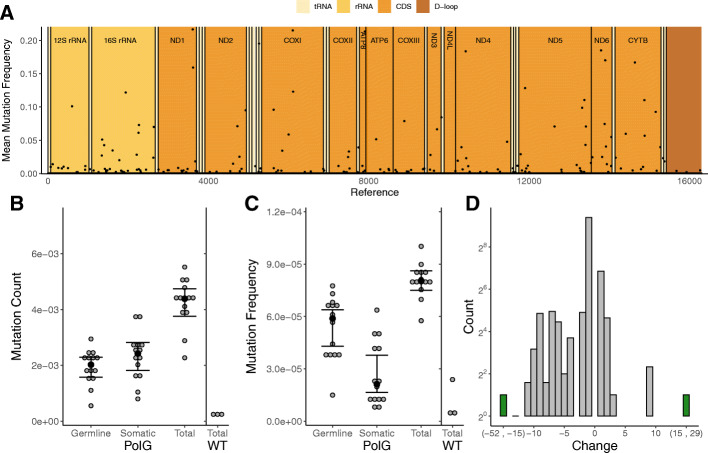
Indels. **A**. The mean mutation frequency for indels is plotted across the entire mtDNA genome. Frequencies were averaged if more than one mutation appeared in one base pair, Normalized values were found using {(x/max(x))}. Germline and somatic indel mutations in liver tissue of PolG mice for both **B**, mutation count, and **C**, mutation frequency. Total is the sum of germline and somatic indels. *N* = 14 for PolG, *N* = 3 for WT (wild type). **D.** Histogram of indel base pair changes. Negative values are deletions and positive values are insertions. Graph depicts all animals summed. Green bars represent bins summarizing multiple changes. Error bars are ±95% CI, black dots represent the median

Compared to point mutations, indels were less abundant; they were still spaced throughout the mtDNA, but were less abundant in the D-loop (Fig. 7A). Wild type mice also have a very low indel count and frequency compared to the PolG mice, as an average of 4 indels were called per wild type mouse compared to about 70 per PolG mouse (Fig. 7, 6B, C). Unlike point mutations, there was no significant difference between germline and somatic mutation count (Fig. 7B; *t =* 1.149, *p =* 0.261). Similar to point mutations, somatic indels were found at approximately half of the frequency of germline indels (Fig. 7C; *t* = − 3.951*, p* < 0.001). PolG indels are primarily small, frameshift deletions, with 850 out of the almost 1000 indels being deletions (Fig. 7D). At random, we would expect close to 33% of CDS indels to be a multiple of 3 and not cause frameshifts, yet only 7% of the CDS indels were not frameshift mutations. Overall, indels in PolG mice are more prevalent than wild type mice, but there are fewer indels in PolG mice compared to point mutations, and there is an underrepresentation of non-frameshift indels.

## Discussion

### Selection shapes mtDNA mutations within and between individuals in the PolG mouse

Consistent with previous studies, our reported PolG mutation count was profoundly higher than wildtype mice (Fig. 2) [7, 9]. Although sequencing depth was about 10,000X in the wild type sequencing samples, very few mutations were found using the same mutation detection pipeline as PolG mice, suggesting that there are few false positive mutations from the PolG mice in our dataset. Also consistent with previous findings, liver tissue shows both a higher count and higher frequency of somatic mtDNA mutations compared with brain tissue in PolG mice [7, 9, 18] (Fig. 2). Previously, it has been argued that higher mtDNA mutation measures in liver tissue may be due to the higher turnover rate of liver cells compared to brain cells, as well as higher mtDNA copy number in liver tissue [7, 37, 38]. It is also possible that the relative differences in clonality between liver and brain tissues play a role in mtDNA mutation spread and detection, as brain tissue has a variety of cell types, many of which are post-mitotic, limiting the somatic spread of mtDNA mutations [39]. However, a complementary or alternative explanation to the difference in somatic mtDNA mutations between tissues is a difference in mtDNA selection strength (Fig. 2, Supplementary Fig. 2) (Additional File 1). Some have hypothesized that because brain tissue has higher energetic demands, selection on mitochondrial function should be especially strong in lineages with large brains [40–44]. Others have experimentally provided evidence for a difference in mtDNA mutation and selection between tissues [45–47]. Specifically, one study demonstrated that BALB mouse liver tissue was more prone to propagate mtDNA from a different mouse strain than the cerebral cortex [46]. In a supplementary analysis considering only somatic mutations, liver tissue had a higher missense mutation frequency than brain (*t* = 2.997; *p* = 0.00274) (Additional File 1). This result suggests that mtDNA in brain tissue may be subjected to higher levels of purifying selection than other tissues, and this may contribute to the overall lower frequency of mtDNA mutations in brain tissues, while low mutation counts may be due to lower turnover rates.

Our results suggest that mtDNA mutations in PolG mice undergo detectable selection through the germline. Somatic missense mutations outnumber silent ones, suggesting mutations that affect resulting amino acids and possibly alter protein function are introduced relatively commonly in PolG mice (Fig. 4). However, germline mutations tend to have fewer missense and more silent mutations (Fig. 4), suggesting that PolG mice receive a subset of less harmful mtDNA mutations from their maternal lineage. Our breeding scheme (Additional File 1) likely resulted in several germline mutations being shared among individuals through a common female ancestor (i.e., the exact same mutation was found at a high frequency in brain and liver of at least two individuals). While this may explain some of the individual variation among the mice examined here, shared germline mutations were relatively rare compared to unique germline mutations and do not qualitatively alter our interpretation on selection in PolG mice. The idea of germline selection in the PolG mice is also supported by the phenotype of homozygous negative PolG mice, which only have germline mutations, but do not display premature aging [7, 9, 12]. Our data on PolG mice confirm strong selection on mtDNA across generations.

In addition to germline mtDNA selection in PolG mice, mtDNA exists as a heteroplasmic pool of variants, meaning selection can occur within an individual [31, 32, 48]. Our results examining somatic mutations also support selection within individuals. Somatic missense mutations appear to have an unexpectedly low mtDNA mutation frequency when compared with silent mutations, despite high overall mutation counts of missense somatic mutations (Fig. 4). This result implies that PolG mice suppress the spread of potentially deleterious mutations. We were also able to replicate a previous result on mtDNA mutation codon position, which also supports a role for within-individual selection on mtDNA mutations [49]: While all codon positions have similar mutation counts, mutations in the third codon position spread to a higher frequency (Fig. 4). Mutations in the third codon are more likely to produce a silent change (the “wobble” effect), therefore third codon mutations are likely under comparatively relaxed selection and can spread to higher frequencies. Amino acid changes were primarily hydrophilic to hydrophobic and hydrophobic to hydrophobic changes (Fig. 5C, D; Supplementary Fig. S4-S7) (Additional File 2). This implies that hydrophobic amino acids may be more tolerated in general. Mt-encoded proteins span the inner mitochondrial membrane (IMM) and generally have a high abundance of hydrophobic residues. The introduction of hydrophilic amino acids into a hydrophobic IMM may lead to a weakened electron transport system [50].

Intuitively, frameshift indels may be more deleterious because they would potentially alter many amino acids in a protein product. However, this pattern of selection was not seen in our indel mtDNA mutation results, as there was an overrepresentation of frameshift mutations (Fig. 7). It is possible that frameshift indels produce non-functional protein products that are not inserted into the inner mitochondrial membrane, therefore making it impossible for them to disrupt OXPHOS and be selected against, whereas non-frameshift indels may produce partially functional proteins that integrate into IMM complexes and cause dysfunction.

### Point mutations are rare in the D-loop

Overall, mtDNA mutations are distributed fairly equally throughout the mt genome (Fig. 3) except for the D-loop where mutations are rare. One deviation from this trend is that the tRNA regions were moderately overrepresented in mtDNA frequency, suggesting that mtDNA mutations in this region may spread through reduced selection. Mitochondrial translation in general is thought to be inefficient compared with nuclear translation, so further alterations in translation due to tRNA mutations may be more easily tolerated [51, 52]. In contrast, the D-loop displayed low counts and frequencies of mtDNA mutations in the PolG mouse, as reported previously [18, 21]. This may serve as evidence that the D-loop is particularly sensitive to mtDNA mutations and several studies have reported the significance of D-loop mutations to disease [53, 54]. Interestingly, the trend appears to reverse in wild type mice and humans, with the D-loop containing a higher number of mtDNA mutations than other regions [49]. It has been reported that mutations in the D-loop lower the mtDNA copy number in cancer patients, which is unsurprising because the D-loop, also known as the control region, likely plays a role in mtDNA replication rate [55].

Many studies have examined the molecular processes of mtDNA replication, but the complete mechanism has not been resolved [56]. Both mouse and human mtDNA have two promoters, one for the heavy strand (O_H_), or Guanine-rich strand, and one for the light strand (O_L_) [57]. One hypothesis states that replication of mtDNA begins at the O_H_ and unlike nuclear genome replication, the new mtDNA molecule remains single-stranded until it reaches the O_L_, which is about 11 kb away from the O_H_ [58]. During mtDNA replication, very specific and conserved sequences participate in processes such as RNA primer complementation, G quadruplexes, and triple-stranded displacement loops [59–62]. Though the D-loop does not code for any products, D-loop mutations may be heavily selected against because they simply prevent or slow propagation of the molecule. One limit to our dataset is that short-read technology would not be able to resolve large multimers, which have been observed in the PolG mouse D-loop [22]. It is clear that the D-loop plays a major role in mtDNA replication, but further work into the precise mechanism may reveal the reasons for seemingly disparate results between PolG and wild type mice.

### Base pair substitutions are characteristic of replication errors

Several explanations have been made for how mtDNA mutations might lead to aging phenotypes, including replication errors, ROS, and hydrolysis leading to increased mitochondrial dysfunction [10, 12, 18, 23, 36, 63–65]. Initially, the mitochondrial theory of aging stated that many aging phenotypes were caused by a feedback loop, or mutational vortex, caused by ROS causing mtDNA mutations, causing more ROS, etc. [10, 64]. More recent studies, including our results in PolG mice, question the importance of ROS in mtDNA mutations and aging [18, 64, 65]. Specifically in PolG mice, though their premature aging is exclusively caused by mtDNA mutations, there is little evidence for an increase in ROS [13, 66]. Studies have also failed to link ROS with mtDNA mutations in wild type animals [67–70]. In our results, C to T (G to A) point mutations, which are indicative of replication errors, were by far the most abundant (Fig. 5). Cytosine to thymine changes are likely caused when the polymerase gamma fails to correct errors, as a common GU wobble base pairing would go uncorrected [9, 71]. This makes intuitive sense, as PolG mice have impaired proofreading capacity. Overall, transitions were also more common than transversions (Fig. 5). ROS tends to cause G to T transversions, which were seen at relatively low levels compared to other changes in our study (Fig. 5), supporting previous studies indicating that ROS levels are not elevated in PolG mice [13]. This evidence suggests that pathological phenotypes in PolG mice result from the inability to correct errors and that ROS likely does not play an exacerbating role in the aging of these animals.

### Lack of strand asymmetry of C to T mutations may reveal insight on mtDNA replication and mutation

In mammalian mtDNA, the lagging strand is the H-strand, or the template strand. During replication, much of the lagging strand is single-stranded, which is thought to expose it to  to more DNA damage [56]. Wild type organisms almost uniformly show a C to T bias on the lagging strand of mtDNA [20, 70, 72–74]. This provides a possible mechanism to explain the cytosine depletion on the H-strand and may explain our finding that silent mutations are exceedingly rare for C to T H-strand mutations (Fig. 6B). It is possible that the depletion of cytosine on the H-strand occurred mainly through silent changes, leaving very few possible C to T synonymous changes remaining on the H-strand. Therefore, any C to T changes that do occur on the H-strand in PolG mice in the present study are more likely to be missense.

A previous study on PolG mice mtDNA mutations showed the opposite finding, reporting double the C to T mutations than G to A on the L-strand [18]. However, their findings were likely due to nucleotide composition bias on the L-strand, which has twice as many cytosines than guanines. Our findings support this hypothesis, as we demonstrated that PolG mice show no strand bias of mtDNA point mutations after correcting for nucleotide composition (Fig. 6A). The differences in PolG and wild type strand-asymmetry patterns may lie in the mechanism of mtDNA mutations. As discussed previously, recent work has criticized the traditional idea that most mtDNA mutations are caused by ROS, and the field has shifted its view to emphasize polymerase errors. In the case of the PolG mouse, this is likely true, as the protected, L-strand of mtDNA strand is just as likely to mutate as the vulnerable H-strand, likely because polymerase errors far outnumber ROS-induced mutations (Fig. 6A). However, this finding may suggest that wild type C to T mtDNA mutations arise from hydrolytic deamidations rather than polymerase errors [75, 76]. Base hydrolysis better explains the strand asymmetry observed in wild type organisms in light of lack of asymmetry in the PolG mutation data, but the data may also be explained by a strand-bias of the mtDNA polymerase due to the differential treatment of the strand during replication. Further research should explore these ideas to provide more evidence for mtDNA mutagenesis due to ROS and/or polymerase errors.

## Conclusions

PolG mice are not only a progeroid model organism, they can also serve as an opportune model for examining mtDNA mutation processes and selection on mtDNA. PolG mice show evidence of mtDNA selection between animals as germline mutations passed onto the next generation show fewer changes predicted to affect protein function. Within individuals, somatic mutations that alter protein function are less likely to rise to high frequencies. We also demonstrated that PolG mice do not display strand asymmetry in their mtDNA mutations. Our results contribute to the discussion on the effect of ROS and polymerase errors on mtDNA mutation rate. We also show that point mutations are relatively rare in the D-loop, possibly due to selection or mtDNA replication mechanisms. Finally, this study provides evidence that aging mechanisms in PolG mice may be related to disruption of OXPHOS through the introduction of hydrophilic amino acids.

## Methods

### PolG mice husbandry and sampling

No animal research was done for this study. We used sequences from animals as previously described in Maclaine et al. [7]. Homozygous PolG mice were bred from heterozygous PolG mice. The breeding scheme and littermate information are available in the supplement (Additional File 1). When male mice reached 2 months of age, mice were randomly placed in an exercise group or a sedentary group. The exercise group had free access to a running wheel until perfusion and tissue extraction at 9–12 months of age. Mice were perfused with a sucrose-based solution after intraperitoneal injection. Half of the brain cut down the midsagittal plane and liver tissue were quickly extracted and frozen in isopentane. DNA was extracted using the QiaPrep Mini kit TM. 10% SDS was added to the brain P1 homogenate. Extracted mtDNA was further purified through NspI digestion, cutting mouse mtDNA into 3 approximately equal fragments, which were agarose gel extracted [7].

### MtDNA sequencing and processing

Sequencing was previously described in Maclaine et al. [7]. Next-generation sequencing was performed on an Illumina Hi-seq 4000 using high-depth 150 nucleotide paired-end sequencing. The shotgun libraries were prepared using the Kapa Biosystems Hyper Library construction kit with no PCR amplification. The average sequencing depth was 8200x for the mitochondrial genome.

Two mice were excluded due to having less than 1 million reads in either the liver or brain, while other samples produced just over 1 million to 18 million reads (Supplementary Table 1) (Additional File 1). Exercised and sedentary groups were combined for the purposes of this study due to no significant difference in their aging phenotype or number of mtDNA mutations [7].

In total, fastq files from 14 PolG animals ranging in ages from 9 to 12 months were examined here. Sequences are available via NCBI (BioProject PRJNA723420). Sample accessions are in Supplementary Table 1 (Additional File 1). To compare mtDNA mutations in PolG mice with those from wild type mice, similar sequencing datasets from brain tissue (*N* = 2) and liver tissue (*N* = 3) of wild type, C57BL6/N mice (aged 9 to 10 weeks) were examined using publicly available datasets (PRJNA434306; sequence read archive accession numbers: SRS2971016, SRS2971023, SRS2971032, SRS2971026, SRS2971022).

Raw sequencing reads were processed using Trimmomatic default parameters, aligned with bwa (v.0.7.17) mem against the mouse mm10 genome (Genbank ID: AY172335), and mutations were called using GATK Mutect2 v4.1.7.0 with all default settings in mitochondria mode (Fig. 1A) [77–79]. The primary benefit of mitochondria mode is to account for the artificial breakpoint in circular genomes. We included wild type samples to check for false-positive mutation calls. Since there were on the order of 3 mutations per wild type mouse, we determined that the pipeline was sufficient in filtering false-positive variant calls. A custom python script containing the exact commands to process these sequence datasets can be found on github [80].

### Defining metrics to quantify mtDNA mutations

Because of the heteroplasmic nature of mtDNA mutations, specific metrics are necessary to describe mtDNA variants. We used the same metrics to quantify mtDNA mutations as described previously in Maclaine et al. [7] and Ma [17],though we did not include the early embryonic category that is included in Ma [17]. This consisted of two main distinctions: somatic vs. germline mutations and mutation count vs. frequency [7, 17] (Fig. 1A). Germline mutations were defined as mutations that were present in both liver and brain tissue as these have a higher probability of being inherited. Because some animals in the study were littermates or otherwise related, we acknowledge that some mutations may be shared between mice. Mutations were required to be at the same base and have the same nucleotide change in both tissues of the same animal to be considered germline. We acknowledge that some mutations classified as germline could have arisen independently in both tissues. Somatic mutations were found in either brain or liver tissue and likely were generated de novo instead of being inherited.

MtDNA mutation count reflects the fraction of basepairs in the mtDNA genome which are mutated, while mtDNA mutation frequency is a metric of mtDNA point mutation saturation (Fig. 1B). Essentially, count describes the number of unique mutations per sample, while frequency describes how common a particular variant was in that sample. These metrics were calculated as follows:

$$ \boldsymbol{mtDNA}\ \boldsymbol{count}=\frac{sum\left( number\ of\ bases\ where\ a\  mutation\ was\ detected\right)}{mt\  genome\ length} $$

$$ \boldsymbol{mtDNA}\ \boldsymbol{frequency}=\frac{sum\left( mutated\ fraction\ of\ reads\ for\ each\ base\ pair\right)}{mt\  genome\ length} $$

When mutation counts and frequencies were compared between different regions of the mtDNA (tRNA, rRNA, coding region, and D-loop), they were calculated as relative to the length of the region. Nucleotide strand bias was corrected for by using samtools depth to find the depth at each basepair in each sample. The total depth for each nucleotide was added for each sample, and all other nucleotides were normalized to cytosine (Additional File 8).

### Characterizing mtDNA mutations

Point mutations were classified into 6 categories based on the starting nucleotide and its mutant variant (e.g., C to T vs. G to T). Redundant changes were combined (e.g., C to A is indistinguishable from G to T in our dataset). Effects of mutations on resulting protein amino acid sequences in protein-coding genes were categorized in two ways. First, the vertebrate mitochondrial codon table was used to categorize mutations as silent, missense, or nonsense (resulting in a premature stop codon). Second, amino acid properties (hydrophobic, hydrophilic, acidic, basic, stop) were examined to investigate how amino acid properties were altered in missense and nonsense mutations. The same amino acid property scheme was used as in Ni et al. [18].

The genome was divided into protein-coding sequences (CDS), tRNA genes, rRNA genes, and D-loop regions using the mm10 genome annotations. Figures that show mutation count and frequency across the genome use a rolling mean of 250 bp, normalized with {(x)/(max(x))}. For evaluating indel mutations, frameshift mutations were considered to be mutations that either added or deleted nucleotides in non-multiples of three.

### Statistics

All statistical analyses were performed in R v4.0.0. Figures were made using ggplot2. Paired t-tests were done to compare mutation count and mutation frequency between brain and liver tissue totals. Lme4 and lmer were used for linear modeling. Linear mixed models were run using both mutation count and mutation frequency as dependent variables. A linear mixed model was run in liver mtDNA mutations with germline/somatic status as a fixed effect and animal as a random effect. We used linear mixed models to investigate variables driving variation in mutation count and frequency: region (D-loop, tRNA, rRNA, CDS) or mutation type (silent, missense, nonsense) were used as one fixed effect and germline/somatic was included as another fixed effect. Another linear mixed model considered only missense C to T mutations and hydrophobicity status (animals with zero in any category were excluded). Animal was always included as a random effect. Data were not normally distributed (as revealed by a Shapiro-Wilk test) and were largely normalized by taking the cube root prior to linear modeling. Differences among basepair changes (e.g., C to T vs. G to T), strand asymmetry, and codon position were evaluated using ANOVA and Tukey post-hoc tests. The R code and raw data are provided as supplemental material (Additional Files 2, 3, 4, 5, 6, 7 and 8).

## Supplementary Information

**Additional file 1: Supplementary Tables 1, 2.**

**Additional file 2.** Text file that contains all R code from data processing, statistics, and figures.

**Additional file 3.** Wild Type Point Mutations Dataset. Dataset containing all Wild Type Point mutations. Output from the script “PolG_bcf_to_excel”

**Additional file 4.** Wild Type Indel Mutations Dataset. Dataset containing all Wild Type Indel mutations. Output from the script “PolG_bcf_to_excel”

**Additional file 5.** PolG Point Mutations Dataset. Dataset containing all PolG Point mutations. Output from the script “PolG_bcf_to_excel”

**Additional file 6.** PolG Indel Mutations Dataset. Dataset containing all PolG Indel mutations. Output from the script “PolG_bcf_to_excel”

**Additional file 7.** Coding Status. Dataset containing all mtDNA genes for figures.

**Additional file 8.** Nucleotide Bias information. Csv file with the percent of each nucleotide mapped to each sample

**Additional file 9.** ReadMe. Readme file with information on the mtDNA mutation datasets.

## Data Availability

The datasets generated and/or analyzed during the current study are available in the NCBI SRA repository, https://www.ncbi.nlm.nih.gov/bioproject/PRJNA723420. All relevant datasets are available in the supplementary information.

## Abbreviations

ATP: adenosine triphosphate
C: cytosine
CDS: protein coding sequence
H-strand: mouse heavy mtDNA strand
L-strand: mouse light mtDNA strand
LHON: Leber hereditary optic neuropathy
MERFF: Myoclonic epilepsy with ragged red fibers
mtDNA: mitochondrial DNA
O_H_: mtDNA heavy strand origin of replication
O_L_: mtDNA light strand origin of replication
OXPHOS: oxidative phosphorylation
PolG: PolgD257A
Rd.: sequencing read
Ref: reference genome
ROS: reactive oxygen species
rRNA: ribosomal ribonucleic acid
T: thymine
tRNA: transfer ribonucleic acid

## References

[1] Taylor RW, Turnbull DM. Mitochondrial DNA mutations in human disease [Internet]. Vol. 6, Nature Reviews Genetics. Europe PMC Funders; 2005 [cited 2020 Aug 21]. p. 389–402. Available from: /pmc/articles/PMC1762815/?report=abstract. 10.1038/nrg1606 PMC1762815 15861210

[2] Kabunga P, Lau AK, Phan K, Puranik R, Liang C, Davis RL, Sue CM, Sy RW Systematic review of cardiac electrical disease in Kearns-Sayre syndrome and mitochondrial cytopathy. Vol. 181, International Journal of Cardiology. Elsevier Ireland Ltd; 2015. p. 303–10, DOI: 10.1016/j.ijcard.2014.12.038. 10.1016/j.ijcard.2014.12.038 25540845

[3] Shoffner JM, Lott MT, Lezza AMS, Seibel P, Ballinger SW, Wallace DC. Myoclonic epilepsy and ragged-red fiber disease (MERRF) is associated with a mitochondrial DNA tRNALys mutation. Cell [Internet]. 1990 15 [cited 2020 Aug 21];61(6):931–7. Available from: http://www.cell.com/article/009286749090059N/fulltext 10.1016/0092-8674(90)90059-n 2112427

[4] Wallace DC. Diseases of the mitochondrial DNA [Internet]. Vol. 61, Annual Review of Biochemistry. Annu Rev Biochem; 1992 [cited 2020 Oct 12]. p. 1175–212. Available from: https://pubmed.ncbi.nlm.nih.gov/1497308/10.1146/annurev.bi.61.070192.005523 1497308

[5] Dobler R, Rogell B, Budar F, Dowling DK. A meta-analysis of the strength and nature of cytoplasmic genetic effects [Internet]. Vol. 27, Journal of Evolutionary Biology. Blackwell Publishing Ltd; 2014 [cited 2020 Oct 11]. p. 2021–34. Available from: 10.1111/jeb.12468 10.1111/jeb.12468 25196503

[6] Giles RE, Blanc H, Cann HM, Wallace AD C. Maternal inheritance of human mitochondrial DNA. Proc Natl Acad Sci U S A [Internet]. 1980 1 [cited 2020 Aug 23];77(11 I):6715–9. Available from: https://www.pnas.org/content/77/11/6715 10.1073/pnas.77.11.6715 PMC350359 6256757

[7] Maclaine KD, Stebbings KA, Llano DA, Rhodes JS. Voluntary wheel running has no impact on brain and liver mitochondrial DNA copy number or mutation measures in the PolG mouse model of aging. Zhang J, editor. PLoS One [Internet]. 2020 2 [cited 2020 Aug 23];15(3):e0226860. Available from: 10.1371/journal.pone.0226860 10.1371/journal.pone.0226860 PMC7051064 32119683

[8] Stewart JB, Chinnery PF. Extreme heterogeneity of human mitochondrial DNA from organelles to populations [Internet]. Nature Reviews Genetics. 2020 [cited 2020 Oct 12]. Available from: www.nature.com/nrg 10.1038/s41576-020-00284-x 32989265

[9] Trifunovic A, Wredenberg A, Falkenberg M, Spelbrink JN, Rovio AT, Bruder CE, et al. Premature ageing in mice expressing defective mitochondrial DNA polymerase. Nature [Internet]. 2004 27 [cited 2019 Feb 28];429(6990):417–23. Available from: http://www.nature.com/articles/nature02517 10.1038/nature02517 15164064

[10] Harman D. The Biologic Clock: The Mitochondria? J Am Geriatr Soc [Internet]. 1972 1 [cited 2020 Aug 24];20(4):145–7. Available from: 10.1111/j.1532-5415.1972.tb00787.x 10.1111/j.1532-5415.1972.tb00787.x 5016631

[11] Harman D. Free radical theory of aging: Consequences of mitochondrial aging. Age (Omaha) [Internet]. 1983 Jul [cited 2021 May 10];6(3):86–94. Available from: 10.1007/BF02432509

[12] Safdar A, Bourgeois JM, Ogborn DI, Little JP, Hettinga BP, Akhtar M, et al. Endurance exercise rescues progeroid aging and induces systemic mitochondrial rejuvenation in mtDNA mutator mice. Proc Natl Acad Sci U S A [Internet]. 2011 [cited 2019 Feb 28];108(10):4135–40. Available from: http://www.ncbi.nlm.nih.gov/pubmed/21368114. 10.1073/pnas.1019581108 PMC3053975 21368114

[13] Trifunovic A, Hansson A, Wredenberg A, Rovio AT, Dufour E, Khvorostov I, et al. Somatic mtDNA mutations cause aging phenotypes without affecting reactive oxygen species production. Proc Natl Acad Sci U S A [Internet]. 2005 [cited 2019 mar 1];102(50):17993–8. Available from: http://www.ncbi.nlm.nih.gov/pubmed/16332961. 10.1073/pnas.0508886102 PMC1312403 16332961

[14] Someya S, Kujoth GC, Kim M-J, Hacker TA, Vermulst M, Weindruch R, et al. Effects of calorie restriction on the lifespan and healthspan of POLG mitochondrial mutator mice. Kato T, editor. PLoS One [Internet]. 2017 3 [cited 2020 Aug 23];12(2):e0171159. Available from: 10.1371/journal.pone.0171159 10.1371/journal.pone.0171159 PMC5291490 28158260

[15] Geurts J, Nasi S, Distel P, Müller-Gerbl M, Prolla TA, Kujoth GC, et al. Prematurely aging mitochondrial DNA mutator mice display subchondral osteopenia and chondrocyte hypertrophy without further osteoarthritis features. Sci Rep [Internet]. 2020 Dec 1 [cited 2020 Oct 11];10(1). Available from: /pmc/articles/PMC6987232/?report=abstract. 10.1038/s41598-020-58385-w PMC6987232 31992827

[16] Ross JM, Coppotelli G, Branca RM, Kim KM, Lehtiö J, Sinclair DA, et al. Voluntary exercise normalizes the proteomic landscape in muscle and brain and improves the phenotype of progeroid mice. Aging Cell [Internet]. 2019 6 [cited 2019 Sep 19]; Available from: 10.1111/acel.13029 10.1111/acel.13029 PMC6826127 31489782

[17] Ma H, Lee Y, Hayama T, Van Dyken C, Marti-Gutierrez N, Li Y, et al. Germline and somatic mtDNA mutations in mouse aging. Trifunovic A, editor. PLoS One [Internet]. 2018 [cited 2019 Apr 30];13(7):e0201304. Available from: http://www.ncbi.nlm.nih.gov/pubmed/30040856. 10.1371/journal.pone.0201304 PMC6057648 30040856

[18] Ni T, Wei G, Shen T, Han M, Lian Y, Fu H, et al. MitoRCA-seq reveals unbalanced cytocine to thymine transition in Polg mutant mice. Sci Rep [Internet]. 2015 27 [cited 2020 Aug 23];5(1):12049. Available from: www.nature.com/scientificreports/10.1038/srep12049 PMC4648470 26212336

[19] Uchimura A, Higuchi M, Minakuchi Y, Ohno M, Toyoda A, Fujiyama A, et al. Germline mutation rates and the long-term phenotypic effects of mutation accumulation in wild-type laboratory mice and mutator mice. Genome Res [Internet]. 2015 Aug 1 [cited 2021 May 28];25(8):1125–34. Available from: /pmc/articles/PMC4509997/. 10.1101/gr.186148.114 PMC4509997 26129709

[20] Arbeithuber B, Hester J, Cremona MA, Stoler N, Zaidi A, Higgins B, et al. Age-related accumulation of de novo mitochondrial mutations in mammalian oocytes and somatic tissues. PLoS Biol [Internet]. 2020 [cited 2021 may 28];18(7):e3000745. Available from: 10.1371/journal.pbio.3000745.g001. 10.1371/journal.pbio.3000745 PMC7363077 32667908

[21] Ameur A, Stewart JB, Freyer C, Hagström E, Ingman M, Larsson NG, et al. Ultra-deep sequencing of mouse mitochondrial DNA: Mutational patterns and their origins. PLoS Genet [Internet]. 2011 Mar [cited 2020 Sep 23];7(3):1002028. Available from: /pmc/articles/PMC3063763/?report=abstract. 10.1371/journal.pgen.1002028 PMC3063763 21455489

[22] Williams SL, Huang J, Edwards YJK, Ulloa RH, Dillon LM, Prolla TA, et al. The mtDNA mutation spectrum of the progeroid polg mutator mouse includes abundant control region multimers. Cell Metab [Internet]. 2010 1 [cited 2020 Oct 11];12(6):675–82. Available from: https://pubmed.ncbi.nlm.nih.gov/21109200/10.1016/j.cmet.2010.11.012 PMC3175596 21109200

[23] Kolesar JE, Safdar A, Abadi A, MacNeil LG, Crane JD, Tarnopolsky MA, et al. Defects in mitochondrial DNA replication and oxidative damage in muscle of mtDNA mutator mice. Free Radic Biol Med [Internet]. 2014 [cited 2019 mar 1];75:241–51. Available from: http://www.ncbi.nlm.nih.gov/pubmed/25106705. 10.1016/j.freeradbiomed.2014.07.038 25106705

[24] Ruiz-Pesini E, Mishmar D, Brandon M, Procaccio V, Wallace DC. Effects of Purifying and Adaptive Selection on Regional Variation in Human mtDNA. Science (80- ) [Internet]. 2004 9 [cited 2021 May 28];303(5655):223–6. Available from: www.sciencemag.orghttp://science.sciencemag.org/10.1126/science.1088434 14716012

[25] Li M, Schröder R, Ni S, Madea B, Stoneking M. Extensive tissue-related and allele-related mtDNA heteroplasmy suggests positive selection for somatic mutations. Proc Natl Acad Sci U S A [Internet]. 2015 24 [cited 2021 May 28];112(8):2491–6. Available from: 10.1073/pnas.1419651112, 112, 8, 2491, 2496 10.1073/pnas.1419651112 PMC4345623 25675502

[26] Hill GE. Life eternal in the face of senescence. In: Mitonuclear Ecology. Oxford University Press; 2019. p. 117–42.

[27] Cree LM, Samuels DC, De Sousa Lopes SC, Rajasimha HK, Wonnapinij P, Mann JR, et al. A reduction of mitochondrial DNA molecules during embryogenesis explains the rapid segregation of genotypes. Nat Genet [Internet]. 2008 Feb [cited 2021 May 17];40(2):249–54. Available from: https://pubmed.ncbi.nlm.nih.gov/18223651/10.1038/ng.2007.63 18223651

[28] Wai T, Teoli D, Shoubridge EA. The mitochondrial DNA genetic bottleneck results from replication of a subpopulation of genomes. Nat Genet [Internet]. 2008 23 [cited 2021 May 17];40(12):1484–8. Available from: http://www.nature.com/naturegenetics 10.1038/ng.258 19029901

[29] Visser JA, Durlinger ALL, Peters IJJ, Van Den Heuvel ER, Rose UM, Kramer P, et al. Increased oocyte degeneration and follicular atresia during the estrous cycle in anti-Müllerian hormone null mice. Endocrinology [Internet]. 2007 May [cited 2021 Jun 1];148(5):2301–8. Available from: https://pubmed.ncbi.nlm.nih.gov/17255205/10.1210/en.2006-1265 17255205

[30] De Fanti S, Vicario S, Lang M, Simone D, Magli C, Luiselli D, et al. Intra-individual purifying selection on mitochondrial DNA variants during human oogenesis. Hum Reprod [Internet]. 2017 1 [cited 2021 Jun 1];32(5):1100–7. Available from: http://omim.org 10.1093/humrep/dex051 PMC5850138 28333293

[31] Rand DM. The units of selection on mitochondrial DNA [Internet]. Vol. 32, Annual Review of Ecology and Systematics. Annual Reviews 4139 El Camino Way, P.O. Box 10139, Palo Alto, CA 94303–0139, USA ; 2001 [cited 2021 Jun 1]. p. 415–48. Available from: www.annualreviews.org

[32] Birky CW. On the origin of mitochondrial mutants: evidence for intracellular selection of mitochondria in the origin of antibiotic resistant cells in yeast. Genetics [Internet]. 1973 [cited 2021 Jun 1];74(3):421–32. Available from: /pmc/articles/PMC1212959/?report=abstract. 10.1093/genetics/74.3.421 PMC1212959 4582949

[33] Tam ZY, Gruber J, Halliwell B, Gunawan R. Context-Dependent Role of Mitochondrial Fusion-Fission in Clonal Expansion of mtDNA Mutations. PLoS Comput Biol [Internet]. 2015 1 [cited 2021 Jun 3];11(5):1004183. Available from: http://www.snf.ch 10.1371/journal.pcbi.1004183 PMC4440705 25996936

[34] Twig G, Elorza A, Molina AJA, Mohamed H, Wikstrom JD, Walzer G, et al. Fission and selective fusion govern mitochondrial segregation and elimination by autophagy. EMBO J [Internet]. 2008 23 [cited 2021 Jun 3];27(2):433–46. Available from: https://pubmed.ncbi.nlm.nih.gov/18200046/10.1038/sj.emboj.7601963 PMC2234339 18200046

[35] Kleele T, Rey T, Winter J, Zaganelli S, Mahecic D, Perreten Lambert H, et al. Distinct fission signatures predict mitochondrial degradation or biogenesis. Nature [Internet]. 2021 [cited 2021 Jun 3];593(7859):435–9. Available from: 10.1038/s41586-021-03510-6. 10.1038/s41586-021-03510-6 33953403

[36] Safdar A, Annis S, Kraytsberg Y, Laverack C, Saleem A, Popadin K, et al. Amelioration of premature aging in mtDNA mutator mouse by exercise: the interplay of oxidative stress, PGC-1α, p53, and DNA damage. A hypothesis. Curr Opin Genet Dev [Internet]. 2016 1 [cited 2019 Mar 1];38:127–32. Available from: https://www.sciencedirect.com/science/article/pii/S0959437X16300855?via%3Dihub 10.1016/j.gde.2016.06.011 PMC5592087 27497229

[37] Edwards JL, Klein RE. Cell renewal in adult mouse tissues. Am J Pathol [Internet]. 1961 [cited 2019 mar 3];38(4):437–53. Available from: http://www.ncbi.nlm.nih.gov/pubmed/13725810. PMC1942349 13725810

[38] Magami Y, Azuma T, Inokuchi H, Kokuno S, Moriyasu F, Kawai K, et al. Cell proliferation and renewal of normal hepatocytes and bile duct cells in adult mouse liver. Liver [Internet]. 2002 [cited 2019 may 11];22(5):419–25. Available from: http://www.ncbi.nlm.nih.gov/pubmed/12390477, . 10.1034/j.1600-0676.2002.01702.x 12390477

[39] Frade JM, Ovejero-Benito MC. Neuronal cell cycle: the neuron itself and its circumstances. Cell Cycle [Internet]. 2015 Mar 13 [cited 2021 Aug 29];14(5):712. Available from: /pmc/articles/PMC4418291/. 10.1080/15384101.2015.1004937 PMC4418291 25590687

[40] Aiello LC, Wheeler P (1995). The expensive-tissue hypothesis: the brain and the digestive system in human and primate evolution. Curr Anthropol.

[41] Kuma KI, Iwabe N, Miyata T. Functional constraints against variations on molecules from the tissue level: Slowly evolving brain-specific genes demonstrated by protein kinase and immunoglobulin supergene families. Mol Biol Evol [Internet]. 1995 [cited 2021 2];12(1):123–30. Available from: https://pubmed.ncbi.nlm.nih.gov/7877487/10.1093/oxfordjournals.molbev.a040181 7877487

[42] Grossman LI, Wildman DE, Schmidt TR, Goodman M. Accelerated evolution of the electron transport chain in anthropoid primates [Internet]. Vol. 20, Trends in Genetics. Trends Genet; 2004 [cited 2021 Jun 2]. p. 578–85. Available from: https://pubmed.ncbi.nlm.nih.gov/15475118/10.1016/j.tig.2004.09.002 15475118

[43] Goldberg A, Wildman DE, Schmidt TR, Hüttemann M, Goodman M, Weiss ML, et al. Adaptive evolution of cytochrome c oxidase subunit VIII in anthropoid primates. Proc Natl Acad Sci U S A [Internet]. 2003 13 [cited 2021 Jun 2];100(10):5873–8. Available from: www.pnas.orgcgidoi10.1073pnas.0931463100 10.1073/pnas.0931463100 PMC156294 12716970

[44] Turnbull HE, Lax NZ, Diodato D, Ansorge O, Turnbull DM. The mitochondrial brain: From mitochondrial genome to neurodegeneration [Internet]. Vol. 1802, Biochimica et Biophysica Acta - Molecular Basis of Disease. Elsevier; 2010 [cited 2021 Jun 2]. p. 111–21. Available from: /pmc/articles/PMC2795853/. 10.1016/j.bbadis.2009.07.010 PMC2795853 19647794

[45] Cortopassi GA, Shibata D, Soong NW, Arnheim N. A pattern of accumulation of a somatic deletion of mitochondrial DNA in aging human tissues. Proc Natl Acad Sci U S A [Internet]. 1992 [cited 2021 Jun 4];89(16):7370–4. Available from: /pmc/articles/PMC49711/?report=abstract. 10.1073/pnas.89.16.7370 PMC49711 1502147

[46] Jenuth JP, Peterson AC, Shoubridge EA. Tissue-specific selection for different mtDNA genotypes in heteroplasmic mice. Nat Genet [Internet]. 1997 [cited 2021 4];16(1):93–5. Available from: http://www.nature.com/naturegenetics 10.1038/ng0597-93 9140402

[47] Samuels DC, Li C, Li B, Song Z, Torstenson E, Boyd Clay H, et al. Recurrent Tissue-Specific mtDNA Mutations Are Common in Humans. Barsh GS, editor. PLoS Genet [Internet]. 2013 7 [cited 2019 Mar 1];9(11):e1003929. Available from: 10.1371/journal.pgen.1003929 10.1371/journal.pgen.1003929 PMC3820769 24244193

[48] Havird JC, Forsythe ES, Williams AM, Werren JH, Dowling DK, Sloan DB (2019). Selfish Mitonuclear Conflict. Curr Biol.

[49] Stewart JB, Freyer C, Elson JL, Wredenberg A, Cansu Z, Trifunovic A, et al. Strong Purifying Selection in Transmission of Mammalian Mitochondrial DNA. Hurst LD, editor. PLoS Biol [Internet]. 2008 29 [cited 2020 Oct 7];6(1):e10. Available from: 10.1371/journal.pbio.0060010 10.1371/journal.pbio.0060010 PMC2214808 18232733

[50] Lloyd RE, Mcgeehan JE. Structural Analysis of Mitochondrial Mutations Reveals a Role for Bigenomic Protein Interactions in Human Disease. PLoS One [Internet]. 2013 9 [cited 2021 Jun 7];8(7):69003. Available from: www.plosone.org 10.1371/journal.pone.0069003 PMC3706435 23874847

[51] Kim CH, Warner JR. Messenger RNA for ribosomal proteins in yeast. J Mol Biol [Internet]. 1983 25 [cited 2020 Oct 13];165(1):79–89. Available from: https://pubmed.ncbi.nlm.nih.gov/6341608/10.1016/s0022-2836(83)80243-5 6341608

[52] Woodson JD, Chory J. Coordination of gene expression between organellar and nuclear genomes [Internet]. Vol. 9, Nature Reviews Genetics. Nat Rev Genet; 2008 [cited 2020 Oct 13]. p. 383–95. Available from: https://pubmed.ncbi.nlm.nih.gov/18368053/10.1038/nrg2348 PMC4854206 18368053

[53] Sharma H, Singh A, Sharma C, Jain SK, Singh N. Mutations in the mitochondrial DNA D-loop region are frequent in cervical cancer. Cancer Cell Int [Internet]. 2005 Dec 16 [cited 2020 Aug 24];5:34. Available from: /pmc/articles/PMC1352382/?report=abstract. 10.1186/1475-2867-5-34 PMC1352382 16359547

[54] Lin J-C, Wang C-C, Jiang R-S, Wang W-Y, Liu S-A. Impact of Somatic Mutations in the D-Loop of Mitochondrial DNA on the Survival of Oral Squamous Cell Carcinoma Patients. Chu P-Y, editor. PLoS One [Internet]. 2015 23 [cited 2020 Aug 24];10(4):e0124322. Available from: 10.1371/journal.pone.0124322 10.1371/journal.pone.0124322 PMC4408030 25906372

[55] Yu M, Zhou Y, Shi Y, Ning L, Yang Y, Wei X, et al. Reduced mitochondrial DNA copy number is correlated with tumor progression and prognosis in Chinese breast cancer patients. IUBMB Life [Internet]. 2007 [cited 2021 May 18];59(7):450–7. Available from: https://pubmed.ncbi.nlm.nih.gov/17654121/10.1080/15216540701509955 17654121

[56] Falkenberg M. Mitochondrial DNA replication in mammalian cells: Overview of the pathway [Internet]. Vol. 62, Essays in Biochemistry. Portland Press Ltd; 2018 [cited 2021 Jun 3]. p. 287–96. Available from: /pmc/articles/PMC6056714/. 10.1042/EBC20170100 PMC6056714 29880722

[57] Berk AJ, Clayton DA (1974). Mechanism of mitochondrial DNA replication in mouse L-cells: asynchronous replication of strands, segregation of circular daughter molecules, aspects of topology and turnover of an initiation sequence. J Mol Biol.

[58] Clayton DA. REPLICATION AND TRANSCRIPTION OF VERTEBRATE MITOCHONDRIAL DNA [Internet]. Vol. 7, Annu. Rev. Cell Bioi. 1991 [cited 2021 Jun 3]. Available from: www.annualreviews.org 10.1146/annurev.cb.07.110191.002321 1809353

[59] Kasamatsu H, Robberson DL, Vinograd J. A Novel Closed-Circular Mitochondrial DNA with Properties of a Replicating Intermediate (density-gradient centrifugation/electron microscopy/separated strands/ethidium bromide/mouse DNA). Vol. 68. 1971. 10.1073/pnas.68.9.2252 PMC389395 5289384

[60] Wanrooij PH, Uhler JP, Shi Y, Westerlund F, Falkenberg M, Gustafsson CM. A hybrid G-quadruplex structure formed between RNA and DNA explains the extraordinary stability of the mitochondrial R-loop. Nucleic Acids Res [Internet]. 2012 Nov [cited 2021 Jun 3];40(20):10334–44. Available from: https://pubmed.ncbi.nlm.nih.gov/22965135/10.1093/nar/gks802 PMC3488243 22965135

[61] Chang DD, Clayton DA. Priming of human mitochondrial DNA replication occurs at the light-strand promoter. Proc Natl Acad Sci U S A [Internet]. 1985 [cited 2021 Jun 3];82(2):351–5. Available from: https://pubmed.ncbi.nlm.nih.gov/2982153/10.1073/pnas.82.2.351 PMC397036 2982153

[62] Chang D, W H, D C. Replication priming and transcription initiate from precisely the same site in mouse mitochondrial DNA - PubMed [Internet]. [cited 2021 Jun 3]. Available from: https://pubmed.ncbi.nlm.nih.gov/2411543/10.1002/j.1460-2075.1985.tb03817.x PMC554382 2411543

[63] Bernal GM, Wahlstrom JS, Crawley CD, Cahill KE, Pytel P, Liang H, et al. Loss of Nfkb1 leads to early onset aging. Aging (Albany NY) [Internet]. 2014 [cited 2019 Apr 21];6(11):931–43. Available from: http://www.ncbi.nlm.nih.gov/pubmed/25553648. 10.18632/aging.100702 PMC4276787 25553648

[64] Wei YH, Lu CY, Lee HC, Pang CY, Ma YS. Oxidative damage and mutation to mitochondrial DNA and age-dependent decline of mitochondrial respiratory function. In: Annals of the New York Academy of Sciences. New York Academy of Sciences; 1998. p. 155–70. 10.1111/j.1749-6632.1998.tb09899.x 9928427

[65] Larsson NG. Somatic mitochondrial DNA mutations in mammalian aging [Internet]. Vol. 79, Annual Review of Biochemistry. Annual Reviews ; 2010 [cited 2020 Oct 7]. p. 683–706. Available from: www.annualreviews.org 10.1146/annurev-biochem-060408-093701 20350166

[66] Logan A, Shabalina IG, Prime TA, Rogatti S, Kalinovich A V., Hartley RC, et al. *In vivo* levels of mitochondrial hydrogen peroxide increase with age in mtDNA mutator mice. Aging Cell [Internet]. 2014 [cited 2019 mar 1];13(4):765–8. Available from: http://www.ncbi.nlm.nih.gov/pubmed/24621297. 10.1111/acel.12212 PMC4326952 24621297

[67] Kauppila JHK, Bonekamp NA, Mourier A, Isokallio MA, Just A, Kauppila TES, et al. Base-excision repair deficiency alone or combined with increased oxidative stress does not increase mtDNA point mutations in mice. Nucleic Acids Res [Internet]. 2018 27 [cited 2021 May 18];46(13):6642–9. Available from: https://pubmed.ncbi.nlm.nih.gov/29860357/10.1093/nar/gky456 PMC6061787 29860357

[68] Kauppila TES, Bratic A, Jensen MB, Baggio F, Partridge L, Jasper H, et al. Mutations of mitochondrial DNA are not major contributors to aging of fruit flies. Proc Natl Acad Sci U S A [Internet]. 2018 9 [cited 2021 May 18];115(41):E9620–9. Available from: 10.1073/pnas.1721683115 10.1073/pnas.1721683115 PMC6187118 30249665

[69] Hoekstra JG, Hipp MJ, Montine TJ, Kennedy SR. Mitochondrial DNA mutations increase in early stage Alzheimer disease and are inconsistent with oxidative damage. Ann Neurol [Internet]. 2016 1 [cited 2021 May 18];80(2):301–6. Available from: https://pubmed.ncbi.nlm.nih.gov/27315116/10.1002/ana.24709 PMC4982791 27315116

[70] Kennedy SR, Salk JJ, Schmitt MW, Loeb LA. Ultra-Sensitive Sequencing Reveals an Age-Related Increase in Somatic Mitochondrial Mutations That Are Inconsistent with Oxidative Damage. PLoS Genet [Internet]. 2013 Sep [cited 2021 May 18];9(9):1003794. Available from: www.plosgenetics.org 10.1371/journal.pgen.1003794 PMC3784509 24086148

[71] Kujoth GC. Mitochondrial DNA Mutations, Oxidative Stress, and Apoptosis in Mammalian Aging. Science (80- ) [Internet]. 2005 15 [cited 2019 Mar 1];309(5733):481–4. Available from: 10.1126/science.1112125 10.1126/science.1112125 16020738

[72] Waneka G, Svendsen JM, Havird JC, Sloan DB. Mitochondrial mutations in *Caenorhabditis elegans* show signatures of oxidative damage and an AT-bias. Surtees JA, editor. Genetics [Internet]. 2021 9 [cited 2021 Aug 24]; Available from: 10.1093/genetics/iyab116/6346985 10.1093/genetics/iyab116 PMC8633081 34849888

[73] YS J, LB A, M G, I M, S N-Z, M R, et al. Origins and functional consequences of somatic mitochondrial DNA mutations in human cancer. Elife [Internet]. 2014 [cited 2021 Aug 27];3. Available from: https://pubmed.ncbi.nlm.nih.gov/25271376/10.7554/eLife.02935 PMC4371858 25271376

[74] Itsara LS, Kennedy SR, Fox EJ, Yu S, Hewitt JJ, Sanchez-Contreras M, et al. Oxidative Stress Is Not a Major Contributor to Somatic Mitochondrial DNA Mutations. PLOS Genet [Internet]. 2014 Feb [cited 2021 Aug 27];10(2):e1003974. Available from: 10.1371/journal.pgen.1003974 10.1371/journal.pgen.1003974 PMC3916223 24516391

[75] A R, C G, G P, C S. Asymmetrical directional mutation pressure in the mitochondrial genome of mammals. Mol Biol Evol [Internet]. 1998 [cited 2021 Aug 28];15(8):957–66. Available from: https://pubmed.ncbi.nlm.nih.gov/9718723/10.1093/oxfordjournals.molbev.a026011 9718723

[76] Zsurka G, Peeva V, Kotlyar A, Kunz WS. Is There Still Any Role for Oxidative Stress in Mitochondrial DNA-Dependent Aging? Genes (Basel) [Internet]. 2018 Apr 1 [cited 2021 Aug 28];9(4). Available from: /pmc/articles/PMC5924517/. 10.3390/genes9040175 PMC5924517 29561808

[77] Li H, Durbin R. Fast and accurate short read alignment with Burrows-Wheeler transform. Bioinformatics [Internet]. 2009 15 [cited 2021 May 28];25(14):1754–60. Available from: http://maq.sourceforge.net 10.1093/bioinformatics/btp324 PMC2705234 19451168

[78] Van der Auwera G, O’Connor B. Genomics in the cloud: using Docker, GATK, and WDL in Terra (1st edition). O’Reilly Media.; 2020.

[79] Bolger AM, Lohse M, Usadel B. Trimmomatic: A flexible trimmer for Illumina sequence data. Bioinformatics [Internet]. 2014 1 [cited 2021 May 28];30(15):2114–20. Available from: http://www.usadellab.org/cms/index 10.1093/bioinformatics/btu170 PMC4103590 24695404

[80] kmaclaine/PolG_bcf_to_excel [Internet]. [cited 2021 May 14]. Available from: https://github.com/kmaclaine/PolG_bcf_to_excel

