# Supplementary material for: The mtDNA mutation spectrum in the PolG mutator mouse reveals germline and somatic selection
Source: BMC Genom Data. 2021 Nov 26;22:52. doi: 10.1186/s12863-021-01005-x (PMC8620558; doi:10.1186/s12863-021-01005-x)
Supplement: Supplementary file 1 — Additional file 1: Supplementary Tables 1, 2. [file 12863_2021_1005_MOESM1_ESM.docx]

**The mtDNA mutation spectrum in the PolG mutator mouse** **reveals germline and somatic selection**

Maclaine, Kendra D^1^*, Stebbings, Kevin A^2^, Havird, Justin C^1^

**Supplementary Figures**

| Animal.1 | Animal.2 | Tissue | Reads | Mapped | % Mapped to mt | BioSample accessions |
| --- | --- | --- | --- | --- | --- | --- |
| 11 | a | Brain | 9849724 | 1726973 | 17.53 | SAMN18817133 |
| 11 | a | Liver | 2112916 | 92308 | 4.37 | SAMN18817134 |
| 12 | b | Brain | 7190928 | 1907618 | 26.53 | SAMN18817135 |
| 12 | b | Liver | 10756778 | 747723 | 6.95 | SAMN18817136 |
| 15 | c | Brain | 4382769 | 642137 | 14.65 | SAMN18817137 |
| 15 | c | Liver | 1941403 | 105739 | 5.45 | SAMN18817138 |
| 22 | d | Brain | 4616391 | 160865 | 3.48 | SAMN18817140 |
| 22 | d | Liver | 1846876 | 1502457 | 81.35 | SAMN18817141 |
| 23 | e | Brain | 15628559 | 1917492 | 12.27 | SAMN18817142 |
| 23 | e | Liver | 1256960 | 149108 | 11.86 | SAMN18817143 |
| 24 | f | Brain | 3449104 | 571079 | 16.56 | SAMN18817144 |
| 24 | f | Liver | 1014379 | 636467 | 62.74 | SAMN18817145 |
| C | g | Brain | 1527588 | 300453 | 19.67 | SAMN18817146 |
| C | g | Liver | 2165009 | 430313 | 19.88 | SAMN18817147 |
| G | h | Brain | 3359296 | 89498 | 2.66 | SAMN18817148 |
| G | h | Liver | 7459444 | 465686 | 6.24 | SAMN18817149 |
| J | i | Brain | 9176083 | 3539909 | 38.58 | SAMN18817150 |
| J | i | Liver | 15319077 | 577759 | 3.77 | SAMN18817151 |
| M | j | Brain | 4489514 | 1496258 | 33.33 | SAMN18817153 |
| M | j | Liver | 1534573 | 609584 | 39.72 | SAMN18817154 |
| O | k | Brain | 17813232 | 451667 | 2.5 | SAMN18817155 |
| O | k | Liver | 8349307 | 312046 | 3.74 | SAMN18817156 |
| R | l | Brain | 10210128 | 2825843 | 27.68 | SAMN18817157 |
| R | l | Liver | 3900199 | 260847 | 6.69 | SAMN18817158 |
| U | m | Brain | 3291656 | 723532 | 21.98 | SAMN18817159 |
| U | m | Liver | 4752326 | 528962 | 11.13 | SAMN18817160 |
| V | n | Brain | 7223264 | 1758240 | 24.34 | SAMN18817161 |
| V | n | Liver | 2328427 | 537037 | 23.06 | SAMN18817162 |

**Supplementary Table 1**

Sequencing information for each sample. Animal.1 is the animal identity in Maclaine et. Al (2020). Animal.2 is the identifier used in this manuscript. Reads is the total number of reads in the sequencing sample. Mapped is how many reads were mapped to the mt genome.

| Animal ID | Treatment | Genotype | Age (months) |
| --- | --- | --- | --- |
| a.1 | Run | PolG | 11.83 |
| a.6 | Run | PolG | 12.1 |
| a.7 | Run | PolG | 10.47 |
| a.11 | Run | PolG | 10.93 |
| a.12 | Run | PolG | 10.8 |
| a.13 | Run | PolG | 10.8 |
| a.14 | Run | PolG | 10.57 |
| a.15 | Run | PolG | 10.53 |
| a.16 | Run | PolG | 10.37 |
| a.18 | Run | PolG | 9.8 |
| a.19 | Run | PolG | 9.77 |
| a.20 | Run | PolG | 9.73 |
| a.21 | Run | PolG | 9.5 |
| a.22 | Run | PolG | 9.47 |
| a.23 | Run | PolG | 9.4 |
| a.24 | Run | PolG | 8.57 |
| B | Sedentary | PolG | 11.67 |
| C | Sedentary | PolG | 12.1 |
| F | Sedentary | PolG | 11.57 |
| G | Sedentary | PolG | 10.47 |
| H | Sedentary | PolG | 11.5 |
| I | Sedentary | PolG | 11.27 |
| J | Sedentary | PolG | 11.2 |
| K | Sedentary | PolG | 9.1 |
| L | Sedentary | PolG | 10.87 |
| M | Sedentary | PolG | 10.73 |
| O | Sedentary | PolG | 9.97 |
| P | Sedentary | PolG | 9.7 |
| Q | Sedentary | PolG | 9.63 |
| R | Sedentary | PolG | 9.4 |
| S | Sedentary | PolG | 10.2 |
| T | Sedentary | PolG | 9.8 |
| U | Sedentary | PolG | 9.33 |
| V | Sedentary | PolG | 8.5 |

**Supplementary Table 2**

Running/Sedentary status, age, and genotype for each animal

| **Animal** | **littermate** |
| --- | --- |
| **a** |  |
| **b** |  |
| **c** | litter 3 |
| **d** | litter 4 |
| **e** | litter 1 |
| **f** | litter 2 |
| **g** |  |
| **h** |  |
| **i** |  |
| **j** | litter 3 |
| **k** |  |
| **l** | litter 4 |
| **m** | litter 1 |
| **n** | litter 2 |

**Supplementary Table 3**

Littermate information for each mouse. For example, animal e and m are littermates. This does not include non-littermate siblings.

**Supplementary Figure 1. (Brain)**

**A**. The mean mutation frequency is plotted across the entire mtDNA genome. Frequencies were averaged if more than one mutation appeared in one base pair, Normalized values were found using {(x/max(x))}.  Lines show the rolling mean (250bp) of both the frequency and count. Mutation Count, **B, C.**, and Mutation Frequency, **D, E.** are shown for protein coding (CDS), tRNA, rRNA, and D-loop regions. Mutation count and frequency are normalized to region length. N=14. Error bars are ± 95% CI, large dots represent the median.

**Supplementary Figure 2. (Brain)**

Mutation Count**, A, B.**, and Mutation Frequency, **D, E.** are shown for silent, missense, and nonsense mutations in the protein coding regions. The mutation count **C,** and frequency, **F,** are shown for each codon position. N=14. Error bars are ±

95% CI, large dots represent the median.

**Supplementary Figure 3. (Brain)**

Mutation count, **A**., and Mutation frequency, **B.**, for each mutation change. Redundant changes are combined. N=14. Error bars are ± 95% CI, dots represent the median. Heatmap showing the coding regions only for **C.,** count and **D.**, frequency. The reference is on the right side and the mutation is across the top. The four types of mutations that showed a significant number of mutations are present. Redundant changes are still combined. Only nonsynonymous mutations are included in the heatmap.

**Supplementary Figure 4.**

Heatmap showing effects of nonsynonymous mutations on amino acid properties for brain tissue mutation count. The reference amino acid state is on the right side and the mutated state is across the top. The four types of mutations that showed a significant number of mutations are shown. CA represents C to A (G->T) respectively. Animal is on the left. White squares are present if there were no mutations. Redundant changes are combined.

**Supplementary Figure 5.**

Heatmap showing effects of nonsynonymous mutations on amino acid properties for liver tissue mutation count. The reference amino acid state is on the right side and the mutated state is across the top. The four types of mutations that showed a significant number of mutations are shown. CA represents C to A (G->T) respectively. Animal is on the left. White squares are present if there were no mutations. Redundant changes are combined.

**Supplementary Figure 6.**

Heatmap showing effects of nonsynonymous mutations on amino acid properties for brain tissue mutation frequency. The reference amino acid state is on the right side and the mutated state is across the top. The four types of mutations that showed a significant number of mutations are shown. CA represents C to A (G->T) respectively. Animal is on the left. White squares are present if there were no mutations. Redundant changes are combined.

**Supplementary Figure 7.**

Heatmap showing effects of nonsynonymous mutations on amino acid properties for liver tissue mutation frequency. The reference amino acid state is on the right side and the mutated state is across the top. The four types of mutations that showed a significant number of mutations are shown. CA represents C to A (G->T) respectively. Animal is on the left. White squares are present if there were no mutations. Redundant changes are combined.

**Supplementary Figure 8. (Brain)**

**A**. The mean mutation frequency for indels is plotted across the entire mtDNA genome Frequencies were averaged if more than one mutation appeared in one basepair, Normalized values were found using {(x/max(x))}.  Lines show the rolling mean (250bp) of both the frequency and count. Germline (mutations which appear in both tissues) and somatic (mutations which appear only in one tissue) indel mutations in brain tissue of PolG mice for both **B**, mutation count, and **C**,mutation frequency**.** Total is the sum of germline and somatic indels. N=14. **D.** Histogram of indel basebair changes. Negative values are deletions and positive values are insertions. Graph depicts all animals summed. Error bars are ± 95% CI, dots represent the median.


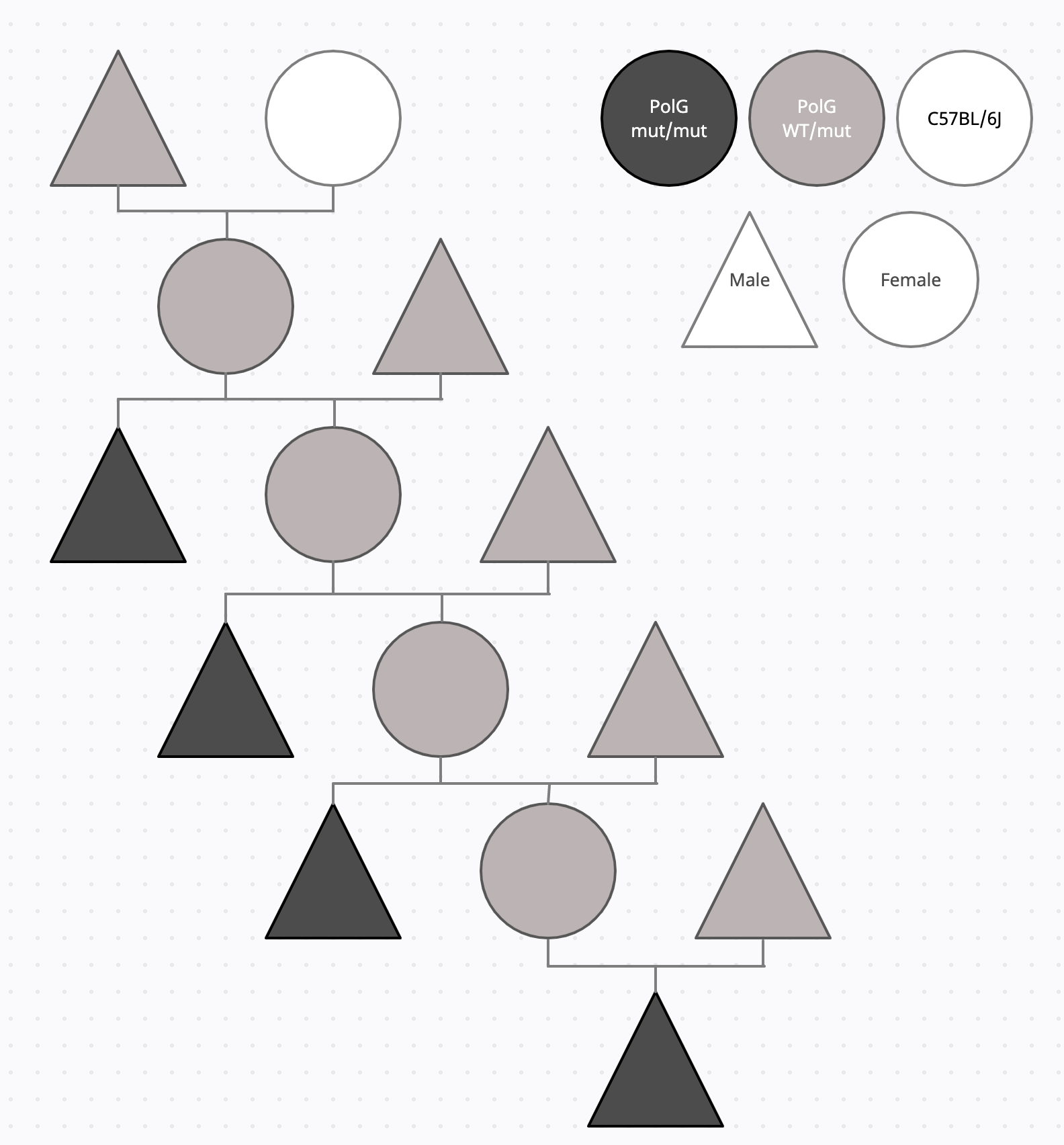


**Supplementary Figure 9.**

Pedigree for PolG mice. Each black triangle represents a possible breeding scheme for a PolG mouse in the study.
